# A realist review of brief interventions for alcohol misuse delivered in emergency departments

**DOI:** 10.1186/s13643-015-0024-4

**Published:** 2015-04-09

**Authors:** Caitlin J Davey, Meredith SH Landy, Amanda Pecora, David Quintero, Kelly E McShane

**Affiliations:** Department of Psychology, Ryerson University, 350 Victoria Street, Toronto, Ontario M5B 2 K3 Canada

**Keywords:** Brief intervention, Realist review, Alcohol, Emergency department

## Abstract

**Background:**

Brief interventions (BIs) involve screening for alcohol misuse and providing feedback to patients about their use, with the aim of reducing alcohol consumption and related consequences. BIs have been implemented in various healthcare settings, including emergency departments (ED), where they have been found to contribute mixed results in their ability to address alcohol misuse among adults. Mechanisms through which BIs work and contextual factors impacting BI effectiveness are not clear. The purpose of this review was to understand how, for whom, and under what circumstances BIs work for adults misusing alcohol and who have been admitted to an ED. A realist review was chosen to answer these questions as realist reviews create context-mechanism-outcome configurations, leading to the development of comprehensive and detailed theories; in this case explaining how and for whom BIs work.

**Methods:**

Databases including PsycINFO, Healthstar, CINAHL, Medline, and Nursing and Allied Health were searched for articles published until December 2013. The search strategy focused on studies examining BIs that targeted alcohol misuse among adults admitted into the ED. The search identified 145 relevant abstracts, of which 36 were included in the review. The literature was synthesized qualitatively (immersion/crystallization).

**Results:**

Four mechanisms were found within reviewed studies, including engagement in/retention of BI materials, resolving ambivalence, increased awareness/insight into consequences of drinking, and increased self-efficacy/empowerment to use skills for change. The following contexts were found to impact mechanisms: emotional state, injury attributed to alcohol use, severity of alcohol use, and baseline stage of change.

**Conclusions:**

This realist review provides advances in theories regarding which mechanisms to target during a BI and which contexts create the most favorable conditions for these mechanisms to occur, ultimately leading to optimal BI outcomes. These results can inform future clinical decision-making when delivering BIs in ED settings. Future research should conduct quantitative examination to confirm these findings.

**Systematic review registration:**

PROSPERO CRD42013006549.

**Electronic supplementary material:**

The online version of this article (doi:10.1186/s13643-015-0024-4) contains supplementary material, which is available to authorized users.

## Background

Alcohol misuse remains one of society’s most challenging and devastating problems [1-3]. It is associated with a range of personal health outcomes, domestic and family violence, and public safety concerns. Brief interventions (BIs) were developed more than 35 years ago and offer a service to address alcohol misuse that is often effective. BIs can be delivered in a variety of clinical settings [4,5]. Drawing heavily from the format and techniques used in motivational interviewing, a BI involves screening for alcohol and substance use, assessing for an alcohol use disorder, presenting the results to the patient, and offering advice and assistance for treatment [6-8]. A commonly used protocol for BIs is the FRAMES model: Feedback, Responsibility, Advice, Menu of options, Empathy, and Self-efficacy [9]. BIs usually take between 5 and 30 min to deliver [10] and, therefore, require only modest time and financial resources [11]. Although a number of systematic reviews have been conducted that document the effectiveness of BIs (for example, [12,13]), how and why such interventions are effective remains unclear. As well, understanding the contextual factors that impact BI effectiveness is an area largely unexplored. To begin to answer such questions, a realist review was conducted with the aim of understanding contextual factors and mechanisms that impact the outcomes of BIs delivered in EDs.

### Effectiveness of brief interventions in emergency departments

A number of systematic reviews have noted the effectiveness of BIs in primary care settings (for example, [12-14]). Due to the effectiveness of BIs in primary care settings, researchers have investigated what contributes to successful outcomes in these settings. In particular, researchers have empirically examined the effectiveness of BIs in EDs, since there may be unique contextual factors associated with this setting that influence the outcomes of BIs delivered there [15]. Several studies have found BIs delivered in EDs to be effective (for example, [15-17]). In particular, Academic ED [15] found reduced drinking among those in a BI group compared to a control group [15]. Additionally, Schermer *et al*. [18] found BI participation to be the strongest protective factor against driving under the influence (DUI) charges at follow-up [18]. Furthermore, D’Onofrio and colleagues [16] found a significant decrease in alcohol-related consequences and service usage in the BI group compared to the control [16].

The studies above present evidence supporting the effectiveness of BIs in reducing alcohol consumption and related consequences in ED settings; however, the literature contains some inconsistent findings regarding the effect of BIs in this context. In particular, a review on the effectiveness of BIs for adults in EDs found mixed results [19]. The findings of this review are captured below:

### Alcohol consumption

- Some studies found significant reductions in alcohol consumption in the BI group compared to the control group, and other studies found no between-group differences.
- A minority of studies found no significant reductions in alcohol consumption in either the BI or control group at 3- and 6-month follow-up.
- At 12-month follow-up, most studies did not find significant differences between groups with regard to alcohol consumption.

### ED visits

- The majority of studies did not find a significant difference between groups on ED visits at follow-up, although one study found a significant difference between the BI group and the control group with regard to the number of ED visits at 12-month post-BI.
- Most studies found that those who received a BI were significantly less likely than those in the control group to experience an alcohol-related injury in the 6 or 12 months following the BI, but some studies reported no group differences or no decrease in injuries at all.

### Criminal activity

- Several studies found BIs to be effective in reducing illegal activity associated with alcohol use, such as a motor vehicle accident (MVA) involving alcohol use or driving under the influence (DUI); however, a minority of studies did not support these results [19].

In sum, the effectiveness of BIs in ED settings for alcohol consumption and related consequences is unclear. As a result, researchers are left with the challenge of identifying patterns to better understand the nuances contributing to the diverse results. Furthermore, these mixed results leave practitioners and policymakers ill equipped to understand how to best implement such interventions. One group of BI researchers identified the pressing need to open the ‘black box’ of emergency care interventions to better understand how and why changes in alcohol consumption occur ([20], p. 199).

### Rationale for realist review

We propose that a realist review can be an effective method to understand the ‘black box’ of ED interventions, as realist reviews focus on elucidating how, for whom, and under what circumstances an intervention works or does not work [21]. Previous reviews have focused on whether BIs achieve desired outcomes (for example, [15,22,23]). This research is useful for determining whether a particular intervention is effective but does not explain why or how the intervention works. In short, outcome-focused research elucidates neither an intervention’s underlying processes nor the contextual factors that can contribute to or hinder its success. Therefore, the aim of this review was to identify patterns of context and outcomes within the literature and to explain these patterns by the mechanisms through which they occur [24]. Mechanisms are causal processes that occur under particular conditions (that is, contexts), yet they do not invariably occur because a particular context may be necessary and/or other mechanisms, occurring simultaneously, might cancel particular mechanisms out [24,25]. Contextual factors are defined as therapist, patient, or setting characteristics that impact the development of mechanisms [21]. As conditions change over time, the context might reflect aspects of these changes [25]. We have chosen to apply a realist approach to the present review in order to synthesize evidence in a way that elucidates the multifaceted nature of BIs.

### Current review

The primary aim of this review was to populate theories that have been identified in the literature as possibly explaining how BIs work with evidence to adjudicate which theory/theories would drive the explanation of BI processes. Using this theory/theories, we aimed to identify context-mechanism-outcome (CMO) configurations, describing the links between all three elements. This information can be used to understand how, for whom, and under what circumstances BIs can be optimized. This focus was selected to fill a gap in the literature, as there is little information on the processes involved in BIs. As well, it serves as a complementary set of information to parallel the findings from a systematic review of BIs for alcohol use among adults in the ED [19].

## Methods

### Scoping of literature

To develop initial theories explaining the mechanisms and contextual factors that lead to optimal outcomes, we conducted an informal scope of the literature and engaged in an iterative process to begin to understand the area. We began by reviewing *Brief intervention for substance use: A manual for use in primary care* [10], which outlined the stage of change model and used FRAMES (Feedback, Responsibility, Advice, Menu of options, Empathy, and Self-efficacy). To supplement the theories put forth in this manual, we reviewed the stages of change model (for example, people move through a cycle of change; [26]), health beliefs model (for example, increased awareness of risk [27]), social learning theory (for example, people develop skills through the observation of others [28]), and behavioral choice theory (for example, increase preference for long term rewards in contrast to short-term rewards [29]), which were all identified by Heather (n.d.) [30]. Finally, we consulted with key stakeholders and frontline service providers to give us a sense of contextual factors and mechanisms that may play a role in BI effectiveness.

### Selection and appraisal of documents

Initially, a search was conducted that focused on primary care settings, more generally. However, upon beginning the abstraction process, the research team decided to focus on ED settings, since there seemed to be some interesting context-specific impacts on BI outcomes. In the second and final search, relevant studies were identified through online searches of databases (PsycINFO, Healthstar, CINAHL, Medline, and Nursing and Allied Health) until December 2013. Search terms included (1) ‘alcohol screening,’ ‘brief intervention,’ ‘brief alcohol intervention,’ or feedback, (2) alcohol, and (3) ‘emergency department’ or ‘emergency room’. These terms were searched in abstracts of articles. Additionally, reference lists from articles initially identified were scanned for other articles to potentially be included. This search strategy generated a total of 443 titles (141 from Medline, 120 from Healthstar, 67 from CINAHL, 73 from PsycINFO, and 42 from Nursing and Allied Health). After removing duplicates, 165 abstracts were identified for initial review and an equal number of abstracts were assigned to each author for review.

An article was excluded if the study it described failed to meet one or more of the inclusion criteria. Included articles examined BIs for alcohol use among adult populations. Participants were defined as adults if they were between 18 and 65 years of age. We included articles in which some participants were outside of our designated age range as long as the majority of participants were within this age range. A BI was defined as a single session lasting between 5 and 30 min [10]; however, some BIs were as long as 60 min and were included as long as it involved only one session. BIs included screening and feedback with the goals of reducing risky alcohol use or alcohol-related problems. Studies that targeted alcohol and drug use were included when the alcohol and drug results were reported separately. To be included, BIs had to be conducted in an ED setting. During the review process, we decided to exclude studies that included booster sessions following the delivery of a BI if the study did not report the effects of the BI alone. There were very few studies of this nature, and it would have been difficult to tease apart the effects of the BI from those of the booster sessions. Studies that reported the effects of the BI and the booster sessions separately were included, as the BI-related outcomes could be determined. Only articles published in English were included. Although mostly empirical and quantitative articles were found, articles incorporating qualitative designs, process evaluations, case studies, and opinion articles could be included in this review as long as it met the other criteria listed above.

If an article appeared to meet inclusion criteria, its full text was retrieved and reviewed to confirm its inclusion. Sixty-eight full-text articles were retrieved and reviewed. Two authors reviewed one half of the full-text articles and two different authors reviewed the other half. Two authors were assigned to review each full-text article to facilitate an iterative process within each pair in the final exclusion or inclusion of studies in the review. After reference lists were examined, 14 more abstracts were identified for review, of which 11 articles were added to the realist review.

The quality of all the articles was evaluated using the quality appraisal recommendations outlined by Pawson *et al*. [21], which encourages authors to first consider relevance and then consider rigor [21]. Wong *et al*. [24] defined study relevance as contributing to theory building and study rigor as generating data using a method that is credible and trustworthy [24]. Study relevance was rated as low, medium, or high. ‘Low’ was used for studies that did not provide any information on mechanisms or contextual factors, ‘medium’ was used for studies that provided information on either contextual factors *or* mechanisms, and ‘high’ was used for studies that addressed both mechanisms *and* contextual factors. Study rigor was rated as weak, moderate, or strong. ‘Weak’ was used to identify studies that lacked relevant details (for example, statistical information or attrition rate) and lacked an appropriate method/design. ‘Moderate’ was used to identify studies that had equivalent strengths and weaknesses. For example, a study that used an appropriate methodology and design as related to their research questions but did not report key information (for example, attrition rate or effect sizes) or were lacking in their interpretations would be rated as moderate in rigor. ‘Strong’ was used to identify tightly controlled studies (that is, randomized controlled trials; RCTs) that used blind coders and reported important details (for example, sample size, attrition, and recruitment method) and appropriate statistical information (for example, *P* values, effect sizes, and type of statistical test), if relevant. Reviews or qualitative papers could also be considered high in rigor, as long as the method used to generate data was deemed credible. We examined rigor in systematic and meta-analytic reviews using the Preferred Reporting Items for Systematic Reviews and Meta-Analyses [31] and planned to use the Qualitative Research Review Guidelines: RATS (relevance, appropriate method, transparency of procedure, and soundness of interpretations; [32]) to assess the rigor of qualitative papers. If a study’s rigor was deemed weak but its relevance was deemed to be medium or high, the study was included in the review. Studies deemed to be low in relevance were excluded from the review. Two authors were assigned to appraise each full-text article to facilitate an iterative process of the quality appraisals.

### Data extraction, analysis, and synthesis process

Information from articles meeting inclusion criteria were extracted and consisted of four domains: (1) a description of the BI and study condition(s); (2) primary outcomes; (3) contextual factors; and (4) mechanisms or underlying causal factors. This information was extracted because it contributed to the development of CMO configurations. Once candidate theories were identified, they were populated with evidence and refined by abstracting and examining articles through a process of qualitative synthesis. Specifically, immersion/crystallization, a common approach to qualitative data analysis, was used. This approach involved the researchers immersing themselves in the data in order to ‘crystallize’, or confirm, reportable themes [33]. As well, the research team relied on corroborating and legitimizing (that is, the process of confirming themes), which is considered to be a critical step in qualitative research [33]. This process occurred through discussions involving the entire research team, on a bi-weekly basis, about the information being extracted from the articles, as well as how the information supported, failed to support, or added to the hypothesized mechanisms and contextual factors. After relevant information was extracted, the team had several meetings to discuss potential CMO configurations (that is, how contexts affected the operations of each mechanism). These meetings helped formulate the results. CMO configurations were further clarified at the writing stage. Through this process, CMO configurations were finalized and followed the literature very closely.

Given the qualitative nature of realist reviews, the identification of mechanisms is considered to be the starting point for future research [34]. The following discussion presents evidence that may begin to support mechanistic properties, representing the first step in identifying and confirming such mechanisms (that is, theory development; [35]). It should be noted that mechanisms are not mutually exclusive and contribute to one another in a non-linear process to impact the outcomes of a BI [21]. Specifically, we conceptualized mechanisms as wave-like that allow for the firing of other mechanisms. The mechanisms listed below have not been empirically examined according to the standards laid out by Baron and Kenny [36] or Kazdin [35,36]. However, the requirements for realist reviews do not state that mechanisms must be empirically examined in this way.

## Results

### Summary of studies

**Figure 1:**
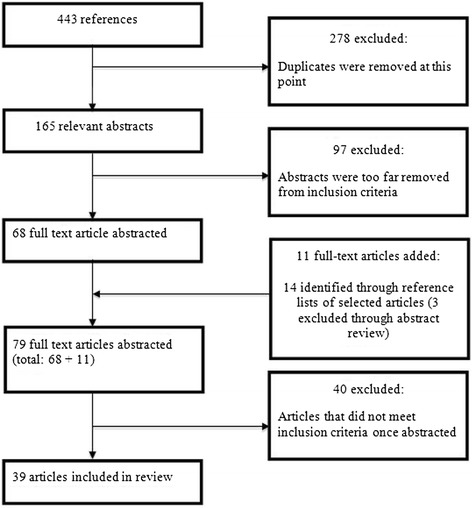
**Article flowchart.**

Thirty-nine studies were included in this review (see Figure 1 for the article flowchart). The majority of the studies included in this review were RCTs (18 articles), followed by pre/post-design studies (17 articles). As well, two reviews, one meta-analysis, and one symposium presentation were included. Twenty-one studies were conducted in the United States (U.S.), 15 were conducted in Europe, one was conducted in Australia, and two review articles included studies conducted in a variety of countries. Most studies compared a BI to a control condition (including standard care [SC] and usual care [UC]; 15 in total). The second most common comparison was of BIs to an active treatment (seven in total). Active treatments included longer feedback (LF), extended counseling (EC), and tailored advice *versus* generic advice. Seven studies did not have a comparison condition. Four articles compared a BI to assessment only. Since several studies included multiple conditions, the total is greater than the number of studies included in the review. See Additional file 1: Table S1 for a description of studies included in the realist review of BIs for alcohol use in the ED.

### Candidate theories

#### Social learning theory

Social learning theory posits that people learn from one another through observation, imitation, and modeling [28]. The theory proposes that there are four conditions through which people learn from others: attention, retention, reproduction, and motivation [28]. Regarding attention, various factors impact the amount of attention that can be paid to a given learning opportunity (for example, affective valence, distinctiveness, complexity, and one’s characteristics; [28]). Retention is the ability to remember what has been learned [28]. Reproduction requires one to reproduce the image using their physical capabilities [28]. Motivation refers to having a good reason to reproduce the learned behavior or skills [28].

#### Health beliefs model

The health beliefs model describes five main perceptions: perceived seriousness, perceived susceptibility, perceived barriers, perceived benefits, and perceived self-efficacy. Perceived seriousness is one’s belief that a health problem is severe [27]. Perceived susceptibility is one’s belief in the likelihood of developing the health problem. The greater the perceived likelihood of developing a health problem, the more likely one will engage in behaviors to decrease this risk [27]. Perceived benefit is one’s opinion of the usefulness of a new behavior in reducing the risk of developing a health problem [27]. Perceived barriers are one’s evaluation of any obstacles that might prevent the adoption of a new health behavior [27]. Perceived self-efficacy has recently been added to this model [37] and refers to one’s belief in their ability to do something [38]. Cues to action are described as influencing health behavior change, including advice from a healthcare provider [39]. Finally, the model described modifying factors, including age, sex, ethnicity, socioeconomic status, and knowledge [27].

#### Stages of change model

The stages of change theory posits that there are five stages that one must go through before change can occur, including precontemplation, contemplation, preparation, action, and maintenance. In precontemplation, the individual is not considering change [26]. In contemplation, the individual is ambivalent about change [26]. In preparation, the individual is ready to change but needs encouragement to make changes. In the action stage, work toward desired behavioral change has been taken. In the maintenance stage, the individual focuses on ongoing and active work to maintain change they have already made. (that is, relapse prevention; [26]). The theory describes specific processes that progress people through the stages of change, including consciousness raising, where one learns new facts, ideas, and tips supporting change [26]. Dramatic relief is another process where the individual experiences negative emotions related to the behavioral risks [26]. Self reevaluation is when one realizes that behavior change is an important part of their identity [26]. Environmental reevaluation is the negative impact of problem behavior on proximal social context [26]. Self-liberation is when one makes a firm commitment to change. Helping relationships is when the individual seeks support for behavior change, and counter conditioning is when there is substitution of a healthy behavior in place of problem behavior [26]. Reinforcement management is an increase in rewards for positive behavior and decrease for negative behaviors [26]. Stimulus control is the removal of reminders or cues to engage in problem behavior and adding cues to engage in new behavior [26]. Social liberation is when one realizes social norms that are changing in the direction supporting the behavior change [26].

There are several criticisms of the stages of change theory: stages might not be distinct from one another, decision-making is not always a conscious process, and change does not always involve planning or preparation [40]. Additionally, it has been argued that the theory might only explain ‘soft outcomes,’ where people move to another stage of change but not necessarily to desired outcomes or behavior change, and the evidence is lacking regarding the effectiveness of stage-matched interventions [40].

#### Behavioral theories of choice

The general premise behind these theories is that preferences for larger, later rewards increase and replace preferences for of smaller, sooner rewards [29]. An example of this theory is behavioral economics, where reinforcements/rewards become substance free [29], but this depends on the relative value of substances compared to other available reinforcers. It is suggested by these theories that substance use continues due to an absence of substance-free reinforcers [29].

### Context-mechanism-outcome configurations

#### Engagement and retention of BI materials

This CMOc includes the mechanism, engaging in, and retaining BI materials, which was found to be necessary in order to achieve desired BI outcomes, as 49% (19/39) [15,16,18,22,41-55] of studies provided some support for the importance of engagement on BI outcomes and none refuted it. All studies supporting engagement were rated as medium or high in relevance and moderate or strong in rigor. Engagement was not explained well by any of our candidate theories. We considered applying social learning theory to explain this mechanism; however, the social part of the theory was not found to be relevant to a BI within the literature reviewed. In conducting a search for theories of engagement and attention, we found that engagement in the context of increased arousal due to ED admission could be better explained through Yerkes-Dodson Law [56], rather than social learning theory. The Yerkes-Dodson Law describes arousal (for example, anxiety, fear, and stress) as contributing to increased attention and motivation, but that this effect eventually plateaus and diminishes once arousal becomes too high [56]. The ED setting, in addition to the crisis that brought the individual into the ED, is thought to create a ‘teachable moment’ which results from the patient’s heightened emotional state upon admission and increased ability to pay attention and learn information from the BI [56]. Emotional state upon ED admission was supported as a contextual factor by 31% (12/39) [15,22,43,46-48,53,54] of studies. Trinks *et al*. [52] suggested that, when ED staff work with many patients over a short period of time or when the ED is very crowded, this can create a chaotic environment [46]. As a result, patients might have difficulty focusing on the information delivered during the BI (arousal might be too high and, therefore, decrease engagement; [56]). Murray *et al*. [49] echoed this possibility [43]. Trinks *et al*. [53] also suggested that the ED might be responsible for change, independently of the BI [47]. This idea needs to be further examined to confirm whether the mechanisms are developed independently of BIs. All studies discussing emotional state upon admission were rated as medium or high in relevance and moderate or strong in rigor. This CMO was hypothesized to be specific to an ED setting, as it is more likely that emotions will be high in this context. When engagement is achieved, it leads to the following outcomes: increased motivation to change (for example, [47]), decreased alcohol consumption (for example, [46]), and decrease alcohol-related consequences (for example, alcohol-related injuries; [48]). It is proposed that these outcomes will initially occur in a stepwise manner (that is, increased motivation, then decreased alcohol use, and then decreased alcohol-related consequences) and then reinforce one another in a non-linear fashion. The engagement mechanism will be impacted by contextual factors outlined above and, therefore, the outcomes listed will also be impacted by the presence of such contexts. It should be noted that it is unclear whether there are certain contexts and mechanisms that lead to particular outcomes, as this has not yet been reported in the current literature.

In addition to emotional state upon ED admission, the engagement mechanism was found to include three other subcomponents representing contexts that influence whether patients will engage in a BI: severity of alcohol use, readiness to change, and presence of injury upon admission. It is theorized that individuals who are admitted to an ED setting and are drinking at moderate severity are most likely to engage in a BI (that is, pay attention and retain the material provided), and 21% (8/39) [15,50,55,57-61] of studies provided some support for this idea. Therefore, this context can impact BI outcomes, including an increase in motivation to change (for example, [59]), a decrease in alcohol consumption (for example, [50]), and a decrease in alcohol-related consequences (for example, [55]). It is hypothesized that those who are drinking at mild or severe levels may be less likely to engage in the BI because they (a) disagree about having any problems with drinking (that is, mild severity) or (b) are dependent on alcohol (that is, severe alcohol use) and, therefore, require more intensive interventions [55]. Mild and severe drinking levels introduce additional CMO configurations, as both will prevent engagement and retention in a BI, but rather elicit a different mechanism: denial of alcohol use risk. Such a CMO configuration will prevent the outcomes of BIs that are listed above (that is, increased motivation to change, decreased alcohol consumption, and decreased alcohol-related consequences). This contextual factor can be partially explained by the health beliefs model, since patients who are drinking at mild severity will likely perceive their risk and susceptibility to health concerns related to alcohol use and the benefit of behavior change as low, resulting in zero engagement in the BI. For those using alcohol at severe levels, perceived benefit of behavior change will also be low, as they might be receiving a lot of perceived benefit from alcohol use, while barriers to change will likely be perceived as high for individuals with severe levels of alcohol use (that is, due to a lack of coping skills and resources). It is theorized that those who do not see the relevance in the BI for the reasons discussed above will be less likely to engage and retain BI information. Although the health beliefs model does not discuss the concepts of engagement or attention, it can help to explain how severity of alcohol use might impact engagement, when combined with the Yerkes-Dodson Law. Specifically, when patients perceive a risk in their alcohol use, as impacted by moderate alcohol use, their arousal levels are likely to rise and are more likely to engage and learn from the BI. We propose that elements of the Yerkes-Dodson Law [56] and the health beliefs [27] model be merged to explain how BIs work, specifically, the link between of engagement in a BI with severity of alcohol use.

Stage of change prior to receiving a BI was found to impact engagement, and 39% (15/39) [41,42,44-47,50,54,58-60,62-65] of articles provided some support for this idea. Only 1% (2/39) [16,48] did not present support for this context. Leontieva *et al*. [47] suggested that individuals in the pre-contemplation stage of change might require more time to achieve desirable outcomes relative to individuals in the contemplation and action phases [47]. It is likely that, if a patient is already motivated to change before receiving the BI, they will be more likely to engage in the intervention, as it will be more applicable to the goals that they will already have for themselves (that is, to decrease alcohol use) and the experience of receiving a BI will increase arousal. Yerkes-Dodson Law describes an increase in focus on motivation that results from arousal [56] and, therefore, stage of change prior to BI implementation will be likely to impact engagement in the BI, when present in combination with (or as a result of) an increased emotional state upon ED admission. Seven studies (18%) specifically linked a ‘teachable moment’ (that is, heightened emotional state) with willingness/readiness/motivation to change [18,22,46,49-51,61]. Korcha *et al*. [46] suggested that the traumatic event that brings patients to the ED might create stress and prime them to reflect on how they got there, increasing their readiness to change at baseline [46] and, as a result, patient engagement. Severity of alcohol use might also impact one’s stage of change prior to receiving a BI, as those who are using alcohol at either a mild or severe level will be less likely to enter the BI in a desired stage of change, as they will either have no issue with alcohol use (mild use) or require more intensive treatment (severe use; [66]). Readiness to change (that is, precontemplation or contemplation stage of change), as a contextual factor, can increase engagement to the BI materials and, as a result, lead to desirable BI outcomes, including further increased readiness to change (for example, perhaps moving people to action or maintenance stages of change; [26]), decreased alcohol use (for example, [51]), and decreased alcohol-related consequences (for example, [18]). All studies discussing readiness to change as a context were rated as medium or high in relevance and moderate or strong in rigor, except Wright *et al*. [58], which was rated as weak in rigor.

It is argued that presence of injury attributed to alcohol use upon admission to the ED will increase the likelihood that one will engage in the BI, and 41% (16/39) [22,42,43,51,54,67-69] of articles provided some support for this context and none refuted it. Daeppen *et al*. [62] suggested that the severity of the injury might impact BI effectiveness, such that minor injuries may lower its effectiveness compared to major injuries [62]. All studies discussed above were rated as medium or high in relevance and moderate or strong in rigor, except Budinger [67], which was rated as weak in rigor [67]. In addition to emotional state upon admission and stage of change prior to BI, the Yerkes-Dodson Law can also explain injury upon ED admission, as linked to the mechanism of engagement in and retention of BI material. It is likely that a high emotional state will occur from an injury (as long as the emotional state is not too high), increasing one’s ability to sustain attention and retain information given to them over the course of a BI. Additionally, the health beliefs model could explain the link between this context and engagement. When someone enters the ED with an injury, this can be conceptualized as a cue to action, making it more likely that patients will engage and retain information provided through the BI. In particular, when patients attribute the injury as being due to alcohol use, they will be more likely to perceive alcohol use as contributing to a severe health concern (that is, injury requiring ED admission) and as increasing their susceptibility to injuries. The BI can provide patients with opportunities to perceive reduced alcohol use as beneficial to them and to perceive less barriers to reducing alcohol use through the menu of options portion of the intervention, which can call upon pre-existing coping strategies. Each of these steps in the health beliefs model as applied to the presence of an injury at ED admission can occur through initial engagement in the BI, as is explained by the Yerkes-Dodson Law and, therefore, it is proposed that particular elements of each theory be combined to explain how BI work and under particular contexts. The presence of an injury attributed to alcohol use, as a contextual factor, can increase engagement to the BI materials (as is explained by the Yerkes-Dodson Law and the health beliefs model) and, as a result, is more likely to lead to desirable BI outcomes, including increased readiness to change (for example, [59]), decreased alcohol use (for example, [55]), and decreased alcohol-related consequences (for example, [51]).

#### Resolving ambivalence

This CMO configuration included the mechanism of resolving ambivalence. Murray *et al*. [49] explained that people seek to maximize consistency between beliefs and attitudes and when they experience cognitive dissonance; they are motivated to resolve such inconsistencies [49]. We theorize that cognitive dissonance is a precondition for ambivalence [26], but most of the literature included in this review provided support for ambivalence as a mechanism and, therefore, will be outlined below.

Resolving ambivalence was found to be a necessary mechanism to achieve desired BI outcomes, and 33% (13/39) [15,23,41,46,49,51,52,54,55,57,58,63,70] of studies provided some support for the importance of resolving ambivalence in impacting BI outcomes. None refuted this mechanism. All studies supporting resolving ambivalence were rated as medium or high in relevance and moderate or strong in rigor, except Wright *et al*. [58], which was rated as weak in rigor [58]. Daeppen *et al*. [63] and Smith *et al*. [51] suggested that ‘change talk’ could be an indicator of resolving ambivalence and is demonstrated when patients exhibit speech in favor of change [51,63]. Walton *et al*. [54] noted that BI providers can use the patient’s attribution of injury to alcohol to draw attention to the discrepancy between the patient’s behaviors and health goals to ‘tip the decisional balance’ in favor of reducing alcohol use [54]. This process helps the patient resolve ambivalence about change. Resolving ambivalence is theorized to be necessary to increase motivation to change [26], which is conceptualized as both a contextual factor and an intermediate outcome of BIs in the present review. When ambivalence about change has been resolved, it can lead to the following outcomes: increased motivation to change (for example, [22]), decreased alcohol consumption (for example, [15]), and decreased alcohol-related consequences (for example, alcohol-related injuries; [57]). This mechanism will be impacted by contextual factors outlined below and, therefore, the outcomes listed will also be impacted by the presence of such contexts.

Resolving ambivalence, in addition to the contextual factors that impact this mechanism, can be explained through one of our candidate theories: stages of change model [26]. Regarding the stages of change model, when one enters the preparation stage, they have resolved ambivalence and are now planning and/or ready to make change [26]. Many BIs incorporate a motivational component [10] and, therefore, it is not surprising that this model would map on to one of our mechanistic findings.

From our included studies, we identified resolving ambivalence as including four subcomponents representing the contexts, influencing whether patients will resolve ambivalence in a BI: stage of change at baseline, severity of alcohol use, presence of injury upon admittance, and emotional state upon admittance. These contextual factors, and how they relate to resolving ambivalence, can be explained by two of our candidate theories: stages of change model and health beliefs model.

Stage of change upon admittance was discussed as an important context by 39% (15/39) 41–42,44-47,50,54,58-60,62-65] of studies, and its link to the resolving ambivalence mechanism can be explained through the stage of change model. When patients enter the BI intervention in the contemplation stage of change, they are ambivalent and will, therefore, be more likely to resolve such ambivalence [26]. Resolving ambivalence may also occur when entering a BI in other stages of change, but this is less likely. In particular, when patients are in precontemplation, they are not considering change yet [26]; however, it is possible for them to achieve ambivalence and eventually resolve it, but this will take longer [26]. Additionally, when patients enter the BI in the preparation stage, they would have already resolved ambivalence [26]; however, it is possible for them to revert to precontemplation or contemplation stages of change during the BI from which they could resolve ambivalence [40]. The possibility could apply for those who enter the BI in the action stage, although resolving ambivalence would be less likely [26,40]. Patients are unlikely to enter a BI in the maintenance stage because changes will have already occurred and will be currently maintained [26]. Stage of change is not proposed to be a linear process and, therefore, patients can cycle through various stages. Therefore, it is possible for patients to resolve ambivalence through a BI when entering this intervention in any stage of change; however, this is most likely to occur when patients are in the contemplation stage of change prior to receiving a BI [26]. When patients enter a BI in the contemplation stage of change, they are more likely to resolve any ambivalence they might have about changing their alcohol use and, as a result, are more likely to achieve to desirable BI outcomes, including a further increase in readiness to change (for example, [54]), decreased alcohol use (for example, [46]), and decreased alcohol-related consequences (for example, [18]).

Severity of alcohol was also found to impact one’s ability to resolve ambivalence through a BI because it is highly linked to one’s stage of change prior to the BI. Therefore, the stages of change model can also explain the link between severity of alcohol use and resolving ambivalence. When alcohol use severity is mild, patients are more likely to deny having a drinking problem and, therefore, they will remain in a precontemplation stage (that is, no willingness to change) or they may be stable in the preparation, action, or maintenance stages of change throughout the BI. When alcohol use is severe, patients are more likely to depend on this substance and, therefore, would be less willing to change. It would be more common for those who use alcohol at severe levels to be in a precontemplation stage of change upon BI implementation and remain in this stage due to their need for more intensive treatment [66]. When alcohol use is within the moderate range, patients are more likely to recognize their risky alcohol use and move through stages of change, making it easier to resolve ambivalence about change during the BI [26,66]. When patient’s level of alcohol use is moderate and they are able to resolve ambivalence through participation in a BI, they are more likely to achieve desirable BI outcomes, including a further increase in motivation to change (for example, [59]), decreased alcohol consumption (for example, [55]), and decreased alcohol-related consequences (for example, [51]).

The impact of injury upon ED admission on resolving ambivalence can also be explained by the stages of change model. Independent of the emotional state that may be elicited by the injury and the resulting admission into the ED, the desire to be physically healthy will be discrepant with one’s current injured state, thus tipping the decisional balance toward resolved ambivalence and increasing motivation to change [26], which is an important and desired outcome of a BI. Increased motivation to change can ultimately lead to other desirable BI outcomes, including decreased alcohol consumption (for example, [55]) and decreased alcohol-related consequences (for example, [51]).

The impact of emotional state on resolving ambivalence can be explained by the Yerkes-Dodson Law for the same reason as described above. A high emotional state upon admission can facilitate a greater focus on one’s motivation, which can increase the likelihood of a patient admitted to the ED entering the contemplation stage of change prior to the BI and then resolving ambivalence during the BI. Upon resolving ambivalence during the BI, patients are more likely to further increase their readiness to change their alcohol use (for example, [54]), decrease their alcohol use (for example, [46]), and decrease alcohol-related consequences (for example, [18]).

#### Increased awareness/insight into consequences of drinking

This CMO configuration includes the mechanism, increased awareness, or insight into the consequences of drinking, which was discussed in 18% (7/39) of studies [47,52,54,62,65,70,71]. BIs give patients time to reflect on their drinking and its consequences, and this can lead to the recognition and awareness of a problem [71]. Leontieva *et al*. [44] suggested that those who realized they had an alcohol problem as a result of a BI reduced harmful behaviors (that is, decreased alcohol-related consequences) and drank less (that is, decreased alcohol consumption; [47]). Walton *et al*. [54] stated that increased awareness about the relationship between one’s alcohol use and injury, gleaned from a BI, might contribute to better alcohol-related outcomes [54]. It is also proposed that the development of this mechanism could lead to an outcome of increased motivation to change alcohol use (for example, [59]). This mechanism and the outcomes noted above would be impacted by various contextual factors, which are outlined below.

This mechanism can be explained by stages of change model. This model describes experiential processes including consciousness raising, where an individual’s awareness about the causes, consequences, and cures for a particular problem behavior [40]. BI likely increases awareness or ‘consciousness’ through providing feedback about problematic drinking and comparisons to the average amount that people of the same age and gender consume [10]. The mechanisms found in this review were not adequately captured by the other processes described in the stages of change model; however, these processes could be further examined in the future. In particular, although we did not find much discussion on social environment of patients in the literature reviewed, it is likely that a patient’s ED admission will impact their environmental reevaluation, which is the negative impact of problem behavior on proximal social context [26], and helping relationships, when the individual seeks support for behavior change [26].

From our included studies, we identified increased insight/awareness of consequences of drinking as including four subcomponents representing contexts that influence whether patients will develop this mechanism in a BI: injury upon ED admission, emotional state upon ED admission, alcohol use severity, and motivation to change when entering BI. Walton *et al*. [54] stated that increased awareness about the relationship between one’s alcohol use and injury, gleaned from a BI, might contribute to better alcohol-related outcomes [54]. The presence of an injury upon ED admission might also prime an increase in arousal and, therefore, these two contextual factors (that is, emotional state and injury) are seen as impacting the development of increased awareness/insight into consequences of drinking as a result of a BI. These two contextual factors can increase one’s ability to focus their awareness on their current problematic drinking as well as the consequences of their drinking patterns (for example, an injury), which is explained above through Yerkes-Dodson Law [56]. High emotional state and the presence of an injury will increase the likelihood that patients will develop insight and increased awareness of their drinking patterns through a BI, which will ultimately impact the achievement of the following BI outcomes: increased motivation to change (for example, [59]), decreased alcohol consumption (for example, [55]), and decreased alcohol-related consequences (for example, [51]).

It is hypothesized that severity of alcohol use and motivation to change at baseline will impact insight, which can be explained by the stages of change model. When a patient has mild alcohol use, they may not believe that they have a problem and, therefore, would not need to develop insight into their drinking behavior. If the patient has high levels of alcohol use, it might be difficult for them to acknowledge their problem for fear of having to give it up. In both cases, a patient’s motivation to change at baseline will be low and, therefore, there will be a lack of willingness to raise consciousness, as the theory suggests [26]. If patient drinking levels are moderate and they enter the BI in contemplation stage of change, this will increase the likelihood of developing insight and increased awareness of their drinking patterns through a BI, ultimately impacting the achievement of the following BI outcomes: increased motivation to change (for example, [54]), decreased alcohol consumption (for example, [55]), and decreased alcohol-related consequences (for example, [51]).

#### Perceived self-efficacy/empowerment in skill use

This CMO configuration included the mechanism of perceived increased self-efficacy/empowerment, which was found to be necessary to achieve desired BI outcomes, and 28% (11/39) [18,41,43,45,46,49,51,54,57,63,65] of studies provided some support for the importance of perceived increased self-efficacy/empowerment. None refuted it. All articles examining perceived self-efficacy/empowerment were rated as medium or high in relevance and moderate or strong in rigor. Murray *et al*. [49] noted that patients are empowered through BIs when they are educated about health-related issues, risks, and protective factors, which may contribute to desirable BI outcomes, such as decreased alcohol consumption [49]. Due to BIs being a very short intervention, it is unlikely that new skills are developed [30]; however, patients may be reminded of their pre-existing coping strategies/skills and feel empowered to/efficacious in using them for change. It is proposed that the development of this mechanism can also increase the likelihood of patients achieving additional desirable outcomes, including increased motivation to change (for example, [54]) and decreased alcohol-related consequences (for example, [51]).

Perceived self-efficacy/empowerment can be explained by one of our candidate theories: health beliefs model. The model states that behavior change is more likely to occur when there is perceived efficacy over behavior change as well as a the perception of a lack of barriers that may impede behavior change [27], which is exactly in line with the self-efficacy/empowerment concept, as one needs to believe in their ability use their skills and empowered to do so to control/change their behavior.

From our reviewed studies, we identified perceived self-efficacy/empowerment as including four subcomponents that represent the contexts influencing this mechanism: severity of alcohol use, baseline stage of change, injury at ED admission, and emotional state at ED admission. Regarding severity of alcohol use and baseline stage of change, Daeppen *et al*. [41] suggested that those with mild or severe drinking patterns might feel less pressure to set goals than those with moderate drinking patterns, impacting the development of self-efficacy/empowerment in pre-existing goal-setting skills, in particular [41]. Therefore, BIs might be the most useful after patients reach a certain threshold of hazardous drinking, since they may be more willing to set goals and, from this, feel efficacious/empowered to use this skill for change. It should also be noted that BIs were designed to target moderate alcohol use [10], which has been identified as an important contextual factor of BIs. The health beliefs model can help to explain this CMO, as when someone has mild alcohol use, they will not perceive any risk or susceptibility to harm as a result of alcohol use and so they will not see the need (or feel motivated) or feel empowered to use any skills they might have to reduce their consumption. When patients have severe levels of alcohol use and low motivation to change, these contexts will serve as obstacles to behavior change, contributing to a decreased or no sense of self-efficacy/empowerment to use any skills that they might have to reduce alcohol use. Therefore, moderate alcohol use will increase the likelihood that a patient will increase their perceived self-efficacy/empowerment in pre-existing skills and ultimately lead to desirable BI outcomes, including increased motivation to change (for example, [54]), decreased alcohol consumption (for example, [51]), and decreased alcohol-related consequences (for example, [55]).

It is hypothesized that, depending on the extent of the injury and amount of emotional arousal, these contexts might decrease one’s sense of empowerment/self-efficacy as either context might be perceived as barriers to behavior change (for example, injury is so severe that there are physical limitations to behavior change and emotional state is so high that one has difficulty concentrating on/tapping into their skills), which would be explained by the health Beliefs model (that is, perceived barriers to behavior change). When patients have an injury at admission (but it is not too severe) and are emotionally aroused (but not to the extent of being unable to concentrate), perceived self-efficacy/empowerment are more likely to increase, contributing to desirable BI outcomes, including increased motivation to change (for example, [54]), decreased alcohol consumption (for example, [55]), and decreased alcohol-related consequences (for example, [51]).

### Summary of findings

**Figure 2:**
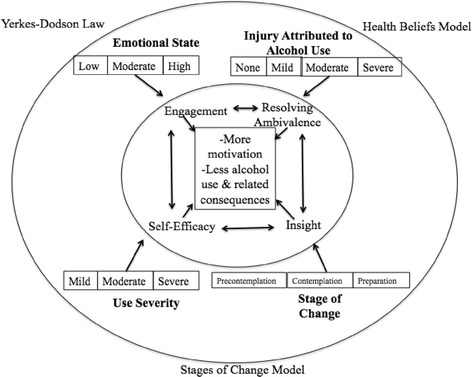
**Context-mechanism-outcome (CMO) configuration.** The CMO configurations were informed by three different theories: Yerkes-Dodson Law, health beliefs model, and the stage of change model. It is argued that aspects of each theory should be used to explain how and under what circumstances BIs work. Specifically, engagement, resolving ambivalence, insight, and self-efficacy were found to be mechanisms leading to desirable BI outcomes, including increased motivation to change, decreased alcohol use, and decreased alcohol-related consequences. Mechanisms are impacted by the following contexts: emotional state upon admission (moderate is best), injury upon ED admission (moderate is best), alcohol use severity (moderate is best), and stage of change at baseline (contemplation is best). See text for more detail.

Through a realist synthesis of the literature, CMO configurations have been discussed and a summary of them are presented below. Engagement in and retention of BI materials, resolving ambivalence, increased insight/awareness of alcohol use/consequences, and perceived self-efficacy were found to be mechanisms through which one can achieve desired BI outcomes (that is, increased motivation to change, reduced alcohol use, and reduced alcohol-related consequences). These mechanisms are theorized to impact one another. There is no evidence supporting any specific mechanism as developing first or last, and we theorize that progression through these mechanisms will not be linear. The following contextual factors were found to impact mechanisms and outcomes: emotional state upon ED admission, injury attributed to alcohol use at ED admission, severity of alcohol use, and baseline stage of change. It is suggested that the Yerkes-Dodson Law, portions of the health beliefs model, and portions of the stages of change model be combined to create a unique theory of BIs, as all three have a role in explaining how, for whom, and under what circumstances BIs work or do not work. See Figure 2 for a visual representation of these CMOs.

### Insufficiently explored contexts and mechanisms

The following contexts were mentioned/examined by very few studies (that is, one to two studies) and/or were not sufficiently explored to draw firm conclusions regarding how they might impact any of the mechanisms found in this review: patient sex/gender, patient age, patient ethnicity, patient marital status, patient education, country in which the BI is implemented, type of ED staff implementing the BI (for example, nurse, physician, and so on), dedication/training/experience of ED staff, and method of BI implementation method (for example, face-to-face, telephone, and so on). It should be noted that the health beliefs model identifies age, sex, ethnicity, personality, socioeconomic status, and knowledge to be ‘modifying factors’; however, it is not clear how these factors modify the other processes identified in the theory, exactly.

Two candidate theories were presented as possibly explaining how BIs work, but no support was found for them: (1) social learning theory and (2) behavioral choice theory. Social learning theory [26] was likely found through our scope of the literature because of the feedback portion of a BI (that is, alerting the patient to how much alcohol people drink on average as compared to their own drinking patters; [10]), which could be considered a way of learning through social means. However, this theory was not supported through this review and this is likely because the focus of this theory is on developing coping skills through the observation of others. It has been suggested that BIs do not increase coping skills, due to the time-limited nature of this intervention [30]. Through our scope of the literature, behavioral choice theory was suggested to apply to a BI because it was thought to move people from focusing on short-term rewards to longer term rewards; however, support for this theory was not found in this review because BIs are likely too short for patients to begin to see the benefit of longer term rewards [29].

## Discussion

The present realist review sought to understand the mechanisms and contextual factors that lead to optimal outcomes for BIs in ED settings. We developed a theory, consisting of CMO configurations, to explain the processes of BIs and contextual factors that contribute to BI effectiveness. This review included 39 articles, which were extracted and synthesized to create the following CMO configurations: (1) engagement in and retention of BI materials, (2) resolving ambivalence, (3) increased insight/awareness, and (4) increased perceived self-efficacy/empowerment in using one’s skills. It is through these mechanisms that patients achieve desirable outcomes from a BI, including an increase in motivation to change, thereby leading to decreased alcohol use and alcohol-related consequences. These processes are more likely to occur when the severity of patients’ alcohol use is moderate, when in contemplation stage of change at admission, and when patients enter the ED with a (moderate) injury attributed to alcohol use and have a heightened (but not too high) emotional state upon ED admission.

To our knowledge, this is the first realist review of BIs in an ED setting and no literature to date has offered CMO configurations on this topic. Due to the novel nature of our work, we were unable to compare our proposed theory to existing explanations. However, the components of each CMO configuration are consistent with theories of learning (that is, Yerkes-Dodson Law; [56]) and behavior change, including the stages of change model [26] and the health beliefs model [27]. Specifically, the link of engagement in/retention of BI materials, resolving ambivalence, increased insight to emotional state upon ED admission, and injury at ED admission was explained by Yerkes-Dodson Law (that is, increase arousal from ED admission or injury increases engagement, focus on motivation, and awareness of problems). The link of engagement to baseline stage of change and severity of alcohol use was also explained through Yerkes-Dodson Law in combination with the health beliefs model (that is, engagement increases through ED admission, which is necessary for someone with moderate alcohol use and in precontemplation stage of change to perceive their use as risky and as increasing their susceptibility to risk due to alcohol use). The stages of change model can explain the link of resolving ambivalence and increased insight to baseline stage of change, alcohol use severity, and injury at ED admission (that is, baseline stage of change will be influenced by severity of alcohol use, impacting both ambivalence and insight, while injury at ED admission can create cognitive dissonance, impacting one’s ambivalence). Finally, the health beliefs model explained the link of self-efficacy to all four contexts (that is, severe use, low motivation to change, high emotional state, and a severe injury could create barriers to perceived empowerment/self-efficacy, but when use is mild, there is no need to feel empowered or efficacious in ability to change). These context-mechanism links increase the likelihood that patients will achieve the following BI outcomes: increased motivation to change, decreased alcohol use, and decreased alcohol-related consequences.

### Strengths, limitations, and future directions

The primary strength of this review was the use of the realist method. The knowledge generated provides heuristic value to the literature, as previous research has not focused on identifying mechanisms and contextual factors that contribute to the success of BIs. A major strength of realist reviews is that the findings can be used to develop and refine theories. As well, the majority of the studies included in this review were deemed to be of high quality (that is, high in relevance and strong in rigor), and only two studies were evaluated as being weak in rigor. When synthesizing the literature and creating the CMO configurations, we considered the quality of each study. All of the studies we included were relevant, as they discussed mechanisms, contextual factors, or both. This was essential in order to answer our research questions. We also ensured that the research team independently screened and extracted each article to reduce bias and increase the accuracy of the findings. We employed a systematic and transparent synthesis process (that is, [33]) and drew heavily on existing literature to create CMO configurations. A strength of the present review is that it included BIs that varied in amount of time, as well as in strategies and frameworks used. Although we were unable to determine how this variability in intervention delivery impacted mechanisms and outcomes of BIs, we recommend that this be examined through future research.

Notwithstanding its aforementioned strengths, our review has several limitations that merit discussion. Our search terms might have limited the articles included in the review, as terms such as ‘emergency department’ and ‘emergency room’ are primarily used in North America, rather than other countries around the world. Furthermore, the majority of studies included in the review were conducted in Western cultures and, therefore, these findings should be applied to other contexts with caution. We were unable to examine mechanisms and most contextual variables (for example, injured patients) in quantitative terms. Although quantitative analysis is not a requirement for a realist review [25,34], we recognize that some may view this as an inherent limitation to the methodology. It should be noted that, within a realist review, quantitative analysis can provide some but not all of the information required to develop a theory. Perhaps due to publication bias, results regarding mechanisms that hinder BI effectiveness were difficult to extract. Since our review was limited to published articles, there was likely a bias toward including articles that found significant results supporting BI effectiveness. Articles that found non-significant or null results for BI effectiveness were less likely to be published, even though such articles might have provided greater insight regarding mechanisms and contextual factors that hinder BI effectiveness.

A final limitation is inherent to the nature of realist reviews. Although employing a realist methodology gives the researcher the opportunity to generate unique and valuable knowledge, there are some limitations to this approach. First, as a result of the multifaceted decision-making process required by this approach, realist reviews are not standardized or reproducible [21]. However, Pawson *et al*. [21] have proposed that this applies to all reviews, including those that follow the *Cochrane Handbook for Systematic Reviews of Interventions* [21]. Therefore, there is considerable transparency with regard to the method employed by the researchers, which makes it clear to readers how the authors arrived at their findings. It is our perspective that the knowledge generated from realist reviews outweighs their limitations. Specifically, realist reviews lead to the development and refinement of models that can be empirically examined and deepen our understanding of how and why interventions work, thereby increasing the effectiveness of interventions. For these reasons, realist reviews are becoming increasingly popular across various fields (for example, [72-74]).

Findings from this realist review highlight the areas of the literature that are relatively developed compared to those that are still in their infancy. Several key areas for future research have been identified. An important area for future research is the quantitative and/or further examination of the theorized CMO configuration. This examination can be used to further refine the theories of what leads to optimal BI outcomes, in turn contributing to improved patient care. In particular, clarification about how gender and age impact BI outcomes is required. Greater understanding of whether these contextual factors impact mechanisms is also needed. From this review, it was clear that BIs were implemented differently from study to study and, therefore, it is recommended that future research expand on the present review and examine how various BI interventions will impact outcomes. Finally, we suggest that treatment processes be better documented to better facilitate an understanding of how and why certain interventions work or do not work.

### Clinical practice considerations

An important clinical implication of this review is that BI providers may want to identify patients’ level of readiness to change before delivering BIs in order to tailor them accordingly. In particular, those who are identified as being in the contemplation stage of change would be more likely to have the necessary mechanisms triggered leading to desirable outcomes. Service providers may also want to assess for severity of substance abuse in order to streamline patients who are alcohol dependent into longer term and more rigorous treatment programs. Those patients identified as using alcohol at a moderate level will be more likely to have the necessary mechanisms triggered leading to desirable outcomes. As well, BI providers might want to consider patient’s emotional state upon BI implementation and whether there is an injury attributed to alcohol use, as those who are anxious (but not overly so) might be primed for a ‘teachable moment’, where a BI could make the most impact. Creating a ‘teachable moment’ might increase a patient’s chances of developing many of the other mechanisms outlined above and ultimately achieve desired BI outcomes. This information may help providers set appropriate expectations regarding which patients are most likely to benefit from treatment.

## Conclusions

The present realist review aimed to elucidate the mechanisms and contextual factors that contribute to optimal outcomes for BIs delivered in an ED setting. We identified two main CMO configurations that can inform future research and clinical decision-making. This review provides valuable information regarding which mechanisms to target during a BI (that is, engagement, ambivalence, insight, and self-efficacy) and which patient characteristics create the most favorable conditions for these mechanisms to occur, ultimately leading to optimal BI outcomes, including increased motivation to change, decreased alcohol consumption, and decreased alcohol-related consequences. The CMO configurations put forth in this review have been populated with evidence from highly relevant and rigorous studies; however, they have not been empirically tested. Testing our proposed CMO configurations is an important next step for research.

## Additional file

Additional file 1: Table S1. Description of studies included in the realist review of BIs for alcohol use in the ED [15-18,22,23,41-55,57-77].
