# Supplementary material for: A realist review of brief interventions for alcohol misuse delivered in emergency departments
Source: Syst Rev. 2015 Apr 9;4:45. doi: 10.1186/s13643-015-0024-4 (PMC4428000; doi:10.1186/s13643-015-0024-4)
Supplement: Additional file 1: Table S1. — Description of studies included in the realist review of BIs for alcohol use in the ED [15-18,22,23,41-55,57-77]. [file 13643_2015_24_MOESM1_ESM.docx]

**Additional file 1: Table S1**

*Description of studies included in the realist review of BIs for alcohol use in the ED*

| Reference; Quality Appraisal  (Relevance and Rigor) | Sample Size (N); Attrition; Setting;  Recruitment Method | Population  Characteristics | Description of Study Conditions; Providers; Training | Details of Study Design  (Design; When Assessed; Measures) | Primary Outcomesa | Contextual Factors | Mechanisms |
| --- | --- | --- | --- | --- | --- | --- | --- |
| Academic ED et al. (2007)  Medium^b^ and  Moderate  Prates et al. (2013)  Medium and  Moderate | -N = 338 (BI) and 361 (control)  **-**21.31% refused,  35.60% dropped out at follow-up  -14 academic EDs across U.S.  **-**Convenience sample | -M age = 36 years  -32% female  -Spanish or English speaking  -Exceeded NIAAA low-risk limits | -BNI: 4 step process  -Control group: screened for at-risk drinking and given printed list of resources  -Physician, nurse, social worker, and emergency medical technician (EMT)  -2 hour training on SBIRT | -Pre/post  -Baseline and 3 months  -CAGE and questions about attending treatment | -BI group had significantly lower levels of typical drinks per week and maximum drinks per occasion  -Prates et al. (2013): Reanalyzed data reported in Academic ED (2007) and found similar results | -At-risk drinkers (CAGE < 2) benefited more from BI than dependent drinkers (CAGE > 2)  -Age and gender had no impact on BI  -Prates et al. (2013): Gender was found to be a predictor of consumption in BI group | -Teachable moment (engagement)  -Cognitive dissonance (connected to resolving ambivalence) |
| Aseltine (2010)  Medium and  Moderate | -N = 190 (BI) and 244 (control)  -21% refused, 62% dropped out (12 months)  **-**14 academic EDs across U.S.  -Convenience sample | **-**M age = 35.8 years  -42% female | **-**BNI: 4 step process  -Control: Screened for at-risk drinking and given a referral list  -Physicians, residents, nurses, EMTs and social workers  -Trained in standardized SBIRT curriculum | -Pre/post  -BNI and control  -Baseline, 3, 6, and 12 months  -NIAAA guidelines | -BI reported significantly fewer drinks per week and maximum drinks per occasion than controls at 3 months; differences no longer significant at 6 and 12 months | -BI efficacy did not differ among risky versus possibly dependent drinkers at any time during follow-up | -None reported |
| Bazargan-Hejazi et al. (2005)  Medium and Strong | -N = 88 (BI) and 97 (control)  -40% refused, 38% dropped out at follow-up  -ED in Los Angeles, CA  -Convenience sample | -M age = 39 years  -20% female  -English or Spanish speaking  -High level of poverty  -Low level of education | -BI: 15-20 minutes; structured  -Control: usual care from ED providers and referral to treatment when requested  -Both conditions received health packet  **-**ED clinicians and peer educators  **-**One month training | -Pre/post  -Baseline and 3 months  **-**AUDIT | -BI and control reduced drinking risk but not significant  -No difference in readiness to change or outcomes for at-risk drinkers | -BI less effective for high-risk drinkers  -High-risk drinkers may not be willing to make change (suggested)  -Gender was not found to predict outcomes | -None reported |
| Blow et al. (2009)  High and  Moderate | -N = 253 (BI) and 241 (control)  -19.2% refused, 14.1% dropped out at follow-up (12 months)  -University affiliated level 1 ED, U.S.  -Convenience sample | -M age = 27.8 years  -29% female  -Acutely injured | **-**BI: Given advice + tailored or generic booklet  **-**Control: No advice + tailored or generic booklet  **-**Research social workers  **-**Training in motivational interviewing | -Pre/post  -Baseline and 12 months  **-**AUDIT-C, DrINC, SIP, and self-report frequency/ quantity of alcohol use | -BI group (tailored and generic booklet) reported significantly lower alcohol consumption at follow-up compared to control group | -BI most effective for more severe alcohol consumption  -BI was helpful for people with an acute injury  -Heightened emotional state at admission positively impacts BI (suggested) | -Teachable moment (engagement) |
| Budinger (2008)  Medium and  Weak | -N = 563 (BI) and 576 (control)  -Attrition was not reported  -ED in U.S.  -Convenience sample | -Ages 18+ (M age not reported)  -Subcritically injured  -Medically stable  -Screened positive for alcohol use disorder | **-**BI: Computer-generated feedback about current alcohol consumption + written information on strategies for behaviour change  **-**Control: Completed initial screening only  **-**Research fellows  **-**Training was not reported | -RCT  -Baseline, 6 and 12 months  -AUDIT-C and AUDIT | -6 months: Compared to control, significantly fewer participants in BI met criteria for at-risk drinking and significantly more participants in BI decreased alcohol intake  -12 months: No differences | -BI was helpful for subcritically injured people | -None reported |
| Crawford et al. (2004)  High and Strong | -N = 287 (BI) and 312 (control)  -8.8% refused, 36% dropped out at follow-up (12 months)  -ED in inner London, England  -Convenience sample | -M age =  44 years  -21.9% female | -BI: Health information leaflet + 30- minute assessment/discussion about current and previous drinking  -Control: Health information leaflet  -ED physicians and  alcohol health workers  -Specialized training and minimum 5 years’ experience | -Single-blind pragmatic RCT  -Baseline, 6 and 12 months  -PAT, Form 90-AQ, TLFB, and Steady Pattern Grid | -6 months: BI drank fewer mean units of alcohol per week than controls; not significant at 12 months | -Women consumed less alcohol than men; lower amounts at baseline associated with lower amounts at follow-up | -Resolving ambivalence  -Developing insight into consequences of drinking |
| Crawford et al. (2010)  High and Strong | -N = 51 (BI) and 52 (control)  -44.9% refused, 28% dropped out at follow-up (6 months)  -ED in central London, England  -Convenience sample | -M age = 37.2 years  -48.5% female  -Patients with deliberate self-harm (DSH) | -BI: Card with time and place of appointment + information leaflet on alcohol and health + 30- minute FRAMES  -Control: Blank card + health information leaflet on alcohol and health  -Alcohol nurse specialist  -Training was not reported | -Single-blind, parallel-group, pragmatic RCT  -Baseline, 3 and 6 months  -Number of subsequent episodes of DSH, AUDIT | -No significant between-group differences found at any time | -BIs may not work for people who deliberately self-harm or who have a personality disorder (suggested) | -Resolving ambivalence |
| Daeppen et al. (2007a)  High and Strong  Daeppen et al. (2010)  Medium and Strong | -N = 134 (objective to decrease/quit drinking), 233 (no objective to decrease/quit drinking)  -19.8% of tapes were lost to follow-up  -See Daeppen et al., (2007a) for setting  -Secondary analysis | -M = 38.4 years  -19.6% female | -BI: Patients dichotomized into 2 groups: (1) expressed objective to decrease/quit drinking and (2) did not express objective to decrease/quit drinking  -Therapist and Training: see Daeppen et al. (2007a) | -Secondary analysis of data in Daeppen et al. (2007a)  -Baseline and 12 months  -AUDIT, weekly alcohol use, heavy drinking episodes per month, MISC (2007b)  -AUDIT, frequency of patient change talk (2010) | -The decrease objective group showed greater reductions in consumption compared to the no decrease group (2007b and 2010)  -12 months: Participants who expressed “toward change” talk drank less than those who expressed “away from change” talk  (2010) | -The decrease objective group had higher levels readiness to change  -Gender or age were not found to impact BI effectiveness  -Those with less severe drinking patterns may feel less pressure to set goals than those with more severe drinking patterns (suggested; 2007b)  -None reported (2010) | -Patients explored ambivalence toward change  -Teachable moment (i.e., engagement)  -Increased self-efficacy (2007b)  -Increased self-efficacy  -Resolving ambivalence (2010) |
| Daeppen et al. (2007b)  Medium and High | -N = 310 (assessment + BI), 342 (assessment only), 335 (control, no assessment)  -17.7% refused, 22% dropped out at follow-up  -Urban academic ED, Lausanne, Switzerland  -Convenience sample | -M age = 36.7 years  -21.8% female  -Injured | -BI: 30 minute assessment + 15 minutes of MI style BI  -Control (1): Assessment + usual care (UC)  -Control (2): UC only  -Master’s level psychologists and nurse  -7-day MI workshop; minimum of 1 year clinical experience + ongoing supervision | -RCT  -Baseline and 12 months  -AUDIT, TLFB, ISS, days hospitalized, % seeking medical or alcohol problem consultation, % patients low-risk drinking at follow-up | -Alcohol use decreased in all groups, no between-group differences  -No between-group differences in seeking medical or alcohol consultation, or in # days hospitalized or absent from work | -Did not find that severity of alcohol use impacted BI outcomes  -Low readiness to change may decrease BI effectiveness (suggested)  -Minor injuries, compared to major injuries, may lower BI effectiveness (suggested) | -None reported |
| Dauer et al. (2006)  High and Strong | -N = 40 (BI) and 45 (control)  -33% dropped out at follow-up (12 months)  -ED of a level I trauma centre, Barcelona, Spain  -Convenience sample | -Mdn age = 26 (no mean reported)  -12% female:  -Injured in motor vehicle accident | -BI: 15-20 minutes, FRAMES  -Control: 5 minutes, empathetic advice  -Nurse and social workers  -5-hour training and regular supervision | -RCT  -Baseline, 3, 6 and 12 months  -AUDIT and AUDIT-C | -No significant between-group differences for any variables at any time point  -Hazardous and binge drinking, as well as traffic violations decreased in both groups | -Hazardous drinkers benefited more from both interventions than non-hazardous drinkers  -BI was beneficial for injured patients  -Most patients were in contemplation stage of change | -Teachable moment (i.e., increased engagement) |
| Desy et al. (2010)  High and Moderate | -N = 26 (BI) and 20 (control)  -51% dropped out  -ED of a level I trauma centre in the UK  -Convenience sample | -M age = 39 years  -40% female  -77% white  -Injured | -BI (SBIRT): 5 to 10 minutes of MI + brochure  -Control (UC): Referrals  -ED Nurse  -Training was not reported | -Pre/post  -Baseline and 3 months  -Medical and driving records | -No significant between-group differences on alcohol consumption, recurring ED visits, or traffic violations | -BI was beneficial for injured patients | -Teachable moment (i.e., engagement in BI)  -Increased self-efficacy |
| D’Onofrio et al. (2008)  High and Strong | -N = 247 (BI) and 247 (control)  -12% refused, 18% dropped out at follow-up (12 months)  -ED of Yale-New Haven Hospital, a tertiary care urban hospital  -Convenience sample | -M age = 34.9 years  -32% female  -Majority white  -AUDIT score >8  -Injured | -BI (BNI): 10 minutes, based on MI  -Control: 1-minute, scripted discharge instructions,  -Physicians, residents, physician associates  -2-hour program with a proficiency test, sessions rated for adherence | -RCT  -Baseline, 6, and 12 months  -TLFB, NIAAA standards, Contemplation Ladder, and Treatment Services Review | -No significant between-group differences  -Alcohol consumption and alcohol-related consequences decreased in both groups  -Service usage increased in both groups | -BI effectiveness was not found to be influenced by severity of alcohol use, readiness to change, or gender  -BI was found to be beneficial for injured patients  -Younger age and being married were associated with decreased alcohol use | -Engagement in BI |
| Forsberg et al. (2000)  Medium and Strong | -N = 80 (BI) and 85 (extended counseling; EC)  -17% refused, 28% dropped out at follow-up (12 months)  -Emergency surgical ward at Danderyd’s hospital in Stockholm, Sweden  -Convenience sample | -Ages: 16 to 73 years (no mean reported)  -52% female  -AUDIT score >8  -Injured | -BI: 26 minutes, assessment and feedback  -EC: Two sessions; assessment and exploration of concerns  -Surgical nurses, surgeons, and psychologists  -Training was not reported | -Pre/post  -Baseline, 6 and 12 months  -TLFB, RTCQ, frequency of alcohol use, average daily amount, sober days, frequency of intoxication, peak amount, and weekly consumption | -Alcohol consumption decreased and readiness to change increased for both groups  -6 months: Greater reductions in peak amounts of alcohol in BI group (not at 12 months) | -BI was found to be beneficial for injured patients | -None reported |
| Gentilello et al. (1999)  High and Strong | -N = 396 (BI) and 396 (control)  -7% refused, 50% dropped out at follow-up (12 months)  -Trauma centre at Harborview Medical Center, University of Washington  -Convenience sample | -M age = 36.1 years  -18% female  -Injured | -BI: 30 minutes, one MI session with personalized feedback, sent letter summarizing session one month later  -Control: Received help for drinking when requested  -Therapist: Psychologists  -Training was not reported | -RCT  -Baseline, 6 and 12 months  -AUDIT, alcohol section of the DIS, SADD, ASI, hospital records of trauma, citations of traffic violations | -Decrease in injuries and trauma treatment in BI group compared to controls  -Significant decrease in alcohol intake for BI group compared to control at 12 months  -BI group had fewer traffic violations than control | -BI most effective for patients with mild to moderate alcohol problems  -BI more effective for patients who were unmarried and unemployed  -BI more effective for females | -Assuming personal responsibility  -Increased self-efficacy and optimism  -Resolving ambivalence |
| Havard, et al. (2007)  Medium and Strong | -N = 10 RCTs (sample sizes ranged from 85 to 1134, M = 582)  -Attrition varied by study  -ED settings not specified  -Recruitment varied by study | -Age varied by study  -% female varied by study  -Injured | -Length of intervention varied from 5-60 minutes | -Meta-analysis  -Quantity/ frequency of drinking,  drinking consequences, alcohol-related injuries  -Commonly used measures: CAGE, AUDIT, PAT | -BIs did not reduce alcohol consumption at 12 months  -6 and 12 months: BI group had lower odds of experiencing an alcohol-related injury (not significant) | -BI was beneficial for injured patients | -None reported |
| Havard et al. (2012)  Medium and Strong | -N = 150 (BI), 154 (control)  -25.1% refused, 19.7% dropped out at follow-up  -EDs in 5 rural communities in South Wales, Australia  -Convenience sample | -Mdn age = 29 years (no mean reported)  -26% female | -BI: Personalized normative feedback mailed to participants  -Control: No feedback  -Therapist: N/A  -Training: N/A | -RCT  -Baseline and 6 weeks  -AUDIT, Quantity/ frequency of alcohol consumption | -BI group consumed significantly less alcohol than the control | -More effective for women than men  -Age, education, baseline alcohol consumption and alcohol dependence did not interact with treatment condition | -None reported |
| Helmklamp et al. (2004)  High and Moderate | -N = Not reported  (article reports on a series of alcohol interventions)  -Attrition: N/A  -ED at West Virginia University Hospital  -Recruitment: N/A | -Age, ethnicity etc. not reported | -Brief MI and counseling sessions tailored to the patient’s readiness to change and the importance of confidence in making changes  -Therapist and Training: N/A | -Summary of results from several studies  -AUDIT | -ED is an appropriate setting for BI programs  -Best for excessive/  binge drinkers through use of behaviour modification and self-directed change | -Patients who think about or take action to change their drinking behaviour may make more significant changes  -Lower readiness to change may make BIs less effective (suggested) | -Teachable moment (engagement)  -High rates acceptance of BIs  -Increased confidence/ empowerment to make a change (self-efficacy) |
| Hungerford et al. (2000)  Medium and Moderate | -N = 83  -22% refused, 64% dropped out at follow-up  -ED at large public hospital in Atlanta, GA  -Convenience sample | -Ages 21 and older (did not report mean)  -45.5% female | -BI: Brief counseling session tailored to patient’s level of motivation to change and severity of alcohol use  -Control: None  -ED staff  -Social work and project-specific training (did not specify) | -Pre/post  -Baseline and 3 months  -AUDIT, Acceptability (calculated rates of consent, received intervention, set goals to change, and reported satisfaction), Likert Scale for readiness to change | -Significant decreases in alcohol intake, alcohol-related harm, and dependence symptoms  -Significant increase in readiness to change | -Dedicated staff  -Intervention tailored to patient’s readiness to change | -Engagement/ acceptability  -Self-efficacy/skill building (goal setting skills) |
| Korcha et al. (2012)  High and Moderate | -N = 162 (not ready), 72 (unsure), and 65 (ready), 152 (intervention)  -Attrition not reported  -ED in Sosnowied, Poland  -Convenience sample | -No mean age reported  -% female not reported | -BI: SBIRT, 15-20 minute  -Control: None  -ED nurses  -Training was not reported | -Pre/post  -Baseline, 3 and 12 months  -RAPS4, Readiness Ruler, SIP, TLFB | -3 months: Ready and unsure groups significantly decreased the quantity of drinks consumed compared to the unsure group | -Heightened emotional state upon admission (suggested)  -Readiness to change impacted effectiveness  -Age did not impact BI effectiveness | -Teachable moment (i.e., engagement)  -Resolving ambivalence |
| Kunz et al. (2004)  Medium and Moderate | -N = 90 (BI) and 104 (EC)  -65% dropped out at follow-up (3 months)  -King Drew Medical Center Emergency department, Los Angeles, CA  -Convenience sample | -M age = 41.5  -19% female  -Most were African American | -BI: Action plans and health information  -EC: Health information  -Health promotion advocates  -Training was not reported | -Pre/post  -Baseline and 3 months  -AUDIT, NIAAA standards | -3 months: BI group consumed less alcohol, had fewer episodes of heavy drinking, and lower AUDIT scores than the control group (not significant) | -Higher motivation to change at baseline had a lower probability of heavy episodic drinking at follow-up  -Gender had no impact on BI | -None reported |
| Leontiva et al. (2003)  High and Moderate | -N = 1304  -40% dropped out at follow-up  -ED in U.S.  -Convenience sample | -Ages 19-29 years (mean not reported)  -% female was not reported  -AUDIT score >5 out of 40 | -BI: Brief MI (four conditions according to stage of change)  -Control: None  -Therapists were not reported  -Training was not reported | -Pre/post  -Baseline and 3 months  -AUDIT | -Baseline stage of change predicted reduction of alcohol intake, harm, and dependency | -Readiness to change impacted BI effectiveness | -Engagement  -Increased awareness/ insight |
| Loftipour et al. (2013)  Moderate and  Weak | -N = 1816  -Attrition not reported  -Tertiary Academic ED in U.S.  -Convenience sample | -Mdn age =43 years (mean not reported)  -Approx. 50% female  -Not dependent  -Spanish speaking | -BI: Computerized alcohol screening and BI (personal feedback, goal setting, and personal alcohol reduction plan)  -Control: None  -Therapists: N/A  -Training: N/A | -Retrospective observational descriptive study  -Baseline and immediately after BI  -Rated readiness to change from 1 to 10 | -Readiness to change increased (did not reported if significant) | -Women had higher readiness to change than men after the BI | -None reported |
| Monti et al. (1999)  High and Strong | -N = 52 (BI) and 42 (Standard Care; SC)  -33% refused, 11% dropped out at follow-up (6 months)  -ED in U.S.  -Convenience sample | -M age = 18.4 years  -36.5% female  -Early adulthood  -Most were white | -BI: 35-40 minutes, used MI principles, given handouts and personalized feedback  -SC: 5 minutes, handout about avoiding drinking and driving, and a list of agencies  -BA to MA-level staff members with 1 to 2 years of experience  -“Extensive training” and weekly supervision, rated for adherence | -RCT  -Baseline, 3, and 6 months  -Young Adult Drinking and Driving Questionnaire, DMV record of moving violations, AIC, Health Behavior Questionnaire, ADQ, Stage of Change Algorithm | -BI group less likely than SC group to have moving violations, drink and drive, alcohol related problems and alcohol-related injuries  -Alcohol use decreased over time, but no significant between-group differences | -Heightened emotional state  -Neither gender nor readiness moderated findings | -Engagement |
| Murray et al. (2010)  High and Moderate | -Parent study N = 551 (BI) 581(control)  -Secondary study N = 137 (physicians) 142 (non-physician)  -29% dropped out (parent study)  -14 academic ED centers in U.S.  -Secondary analysis of data | -M age = 35 years  -32% female  -42% African-American  -English and Spanish speaking | -BI (SBIRT): 10 minute BNI, written low-risk drinking guidelines and treatment centre referrals  -Control: Written low-risk guidelines and treatment referral  -Physicians, nurses, social workers, EMTs  -2-hour interactive SBIRT training | -Pre/post  non-randomized comparison group (parent study) and secondary analysis  -Baseline and 3 months  -NIAAA standards | -BI was not found to be more effective when delivered by a physician compared to a non-physician | -Neither gender nor marital status were found to impact BI effectiveness  -Participants with higher education had less alcohol consumption per day and less alcohol consumption on days used than those with less education  -Heightened emotional state upon admission to the ED (suggested) | -Engagement  -Feeling empowered  -Resolving ambivalence |
| Neumann et al. (2006)  High and Strong | -N = 581 (BI) and 575 (Control)  -39.8% refused, 41.9% dropped out at follow-up (12 months)  -ED of the Charite Campus Mitte, University Hospital of Berlin  -Convenience sample | -M age = 30.5 years  -21% female  -Most were employed  -Most in pre-contemplation stage of change | -BI: Computer-generated feedback about current drinking habits, letter sent summarizing BI, used FRAMES, given alcohol resources  -Control: Routine ED procedures  -Therapists: N/A  -Training: N/A | -Prospective RCT  -Baseline, 6, and 12 months  -British Medical Association (BMA) criteria for at-risk drinking, and AUDIT | -6 and 12 months: Decreased alcohol consumption in BI condition compared to controls  -6 months: Greater decrease in portion meeting BMA criteria for at-risk drinking in BI condition compared to control (not significant at 12 months) | -BI had greatest effect on those in contemplation stage of change  -Younger patients benefited more from BI  -The more alcohol consumption at baseline, the more likely participants were to be at-risk drinkers at 6 months (severity of use)  -Heightened emotional state upon admission to the ED impacts BI effectiveness (suggested) | -Resolving ambivalence  -Engagement in BI  -Insight/ awareness of drinking problem |
| Nordqvist et al. (2005)  High and Moderate | -N = 260 (BI) and 359 (control)  -20% refused, 64% dropped out at follow-up (6 months)  -ED at Motala County Hospital in southern Sweden  -Convenience sample | -M age = 38.75 years  -40% female  -Injured  -Swedish | -BI (Cohort B): Screening  + written advice  -Control (Cohort A): Screening only  -Conditions were also separated by ‘risky’ and ‘non-risky’ drinkers  -Project assistant  -Training was not reported | -Pre/post  -Baseline and 6 months  -AUDIT-C and a readiness to change question | -Control: ‘Risky’ had a decrease in drinking and ‘non-risky’ had all measures increase  -BI: ‘Risky’ had a decrease in drinking and ‘non-risky’ increased in all measures  -The BI group became more ready to change than the control | -Higher baseline consumption was associated with greater decrease at follow-up  -BI was helpful for injured patients | -Increased self-awareness |
| Roudsari et al. (2009)  Medium and Strong | -N = 737 (BI), 756 (control)  -21.3% refused, 40% dropped out at follow-up (12 months)  -Level I urban trauma center in the U.S.  -Convenience sample | -Ages 18+ years (no mean reported)  -Most were white  -% female was not reported  -Injured | -BI: Non-confrontational, patient-centered conversation  -Control: Questionnaire and referral  -Master’s level clinicians and students  -5-day certified training session from the Motivational Interview Network of Trainers | -RCT  -Baseline, 6 and 12 months  -BAC, CAGE, self-reported injuries | -BI  not associated with reduction in risk of general injuries, alcohol-related injuries, or ED-required injuries | -BI did not help reduce injuries among initially injured patients  -Controlled for gender  -No differences for any ethnicities | -None reported |
| Saitz et al. (2006)  High and Moderate | -N = 341 (not broken down by condition)  -Attrition was not reported  -Urban general hospital in U.S.  -Convenience sample | -M age = 44 years  -29% female  -45% Black  -77% alcohol dependent | -Description of intervention, therapists, and training not reported | -Symposium presentation (including RCT by D’Onofrio et al., 2008)  -Outcome measures were not reported | -There were few significant  effects of the intervention on consumption among all subjects with unhealthy alcohol use | -Heightened emotional state upon admission to ED increases BI effectiveness (suggested)  -BI was not sufficient for decreasing consumption among alcohol dependent patients (suggested) | -Engagement in BI |
| Schermer et al. (2006)  High and Moderate | -N = 62 (BI) and 64 (SC)  -19.75% refused (did not report follow-up attrition)  -University of New Mexico Hospital Trauma Centre  -Convenience sample | -M age = 33 years  -30.95% female  -Involved in motor vehicle accident (MVA) | **-**BI: 30 minutes, MI style  **-**Control (SC): Participants received a list of phone numbers with alcohol treatment organization  **-**Social worker or trauma surgeon  **-**Training was not reported | -RCT  -Baseline and 3 years  -Number of DUIs in 3 years following BI | -BI group had fewer DUIs than SC group  -BI was strongest protective factor against a DUI arrest | -Severity of alcohol use was not found to impact DUIs  -Gender was not found to impact BI effectiveness  -Younger age had better BI outcomes  -Heightened emotional state upon ED admission positively impacts BI effectiveness | -Engagement  -Self-efficacy |
| Smith et al. (2003)  High and Strong | -N = 75 (BI) and 76 (control)  -7% refused, 19% dropped out at follow-up (12 months)  -Accident and Emergency department at an urban teaching hospital, UK  -Convenience sample | -M age = 24 years  -0% female  -Facial injury | -BI: MI-informed  -Control: Given a list alcohol resources  -Nurses  -Trained by Clinical Psychologist in two 90-minute workshops, learned style of MI and strategies  -Sessions rated for adherence | -Prospective RCT  -Baseline, 3 and 12 months  -AUDIT, Brief Alcohol Problems Questionnaire, 901 Drink Diary Section, Social Satisfaction Questionnaire | -Less total alcohol consumption, more days abstinent, less hazardous drinkers and less consumption in a week for BI than control at 12 months | -Injured patients benefited from BI  -Heightened emotional state upon ED admission positively impacts BI effectiveness  -Young males benefitted from BI (no comparison to females) | -Engagement in BI  -Resolving ambivalence  -Increased responsibility for change  -Self-efficacy |
| Suffoletto et al. (2012)  High and Strong | -N = 45 (Intervention), 45 (BI), and 45 (control)  -3% refused, 13% dropped out at follow-up  -3 EDs in Western Pennsylvania  -Convenience sample | -M age = 21 years  -66% female  -24% Black | -BI: Control + required to respond to questionnaire  -Intervention: Feedback about alcohol use sent to client by text message each week  -Control: Reminder to complete survey sent by text message  -Research associates  -Training was not reported | -RCT  -Baseline and each week for 12 weeks  -AUDIT-C, TLFB, and RAPI | -Patients willing to set a goal were less likely to experience  heavy drinking days the following week compared with those who did not set goals | -Readiness to change may interact with severity of alcohol use (suggested) | -Insight into consequences of drinking/  increased awareness  -Self-efficacy |
| Trinks et al. (2010)  Medium and Moderate | -N = 52 (Long Feedback; LF), 41 (Short Feedback; SF)  -7% refused, 83.3% dropped out at follow-up  -ED facility  of the Motala County Hospital, Sweden  -Convenience sample | -Ages 18-69 years (no mean reported)  -69% female | -SF: Graphic  illustration of the risks of  weekly alcohol consumption  -LF: Same as SF + tailored  advice and information on weekly alcohol intake level, and assessment of motivation to change drinking patterns  -Therapist: Triage nurses  -Training was not reported | -Prospective RCT  -Baseline and 6 months  -Outcomes measures were not reported | -6 months: Participants in the LF group significantly reduced their heavy episodic drinking compared to participants in the SF group  -SF group reduced heavy episodic drinking somewhat (not statistically significant) | -None reported | -Engagement  -Resolving ambivalence  -Increased insight/ awareness of drinking problem may increase motivation |
| Trinks et al. (2013)  High and Moderate | -N =  -7% refused, 78% dropped out at follow-up  -ED facility  of the Motala County Hospital, Sweden  -Convenience sample | -Ages 18-69 years (no mean reported)  -44% female  -Injured and non-injured | -BI: Completed computerized program and received printed handout with feedback on alcohol use  -Control: None  -Therapists: N/A  -Training: N/A | -Pre/post  -Baseline and 6 months  -Self-reported frequency, quantity, and frequency of heavy episodic drinking  -Asked about reasons for alcohol reduction | -Those influenced by ED were 6X more likely to decrease use  -Those influenced by healthcare provider were 4X more likely to decrease use  -Those motivated to change at baseline were 2X more likely to decrease use | -No differences between those who reduced drinking on age, sex or education  -BI was impacted by ED setting (possibly impacted outcomes independently of BI), healthcare provider, and motivation to change  -Injury and acute drinking levels did not impact the BI  -Heightened emotional state upon ED admission positively impacts BI effectiveness | -Engagement: Those who considered feedback were 4X likely to decrease alcohol use |
| Vaca et al. (2011)  High and Moderate | -N = 385  -27% refused, 43% dropped out at follow-up (6 months)  -Tertiary care  university hospital ED, U.S.  -Convenience sample | -Ages 18+ years (no mean reported)  -35% female  -Injured  -AUDIT score of <19 | -BI: Computer-guided BNI with personalized  feedback, assessed reasons  for cutting down, goal setting, and a printed  personal alcohol reduction plan  -Control: None  -Research associates  Training was not reported | -Pre/post  -Baseline, 1 and 6 months  -NIAAA guidelines and measured readiness to change (measure not reported) | -47%  no longer exceeded  NIAAA limits | -BIs most effective for mild to moderate drinking severity and may interact with readiness to change  -Ages over 30 years benefitted from BI  -No difference in alcohol reduction between injured and non-injured or male and female patients | -Self-efficacy |
| Walton et al. (2008)  High and Strong | -N = 494 (total)  -11% refused, 14% dropped out at follow-up (12 months)  -Level 1 ED, Midwestern U.S.  -Convenience sample | -M age = 27.8 years  -% female was not reported  -Injured in last 24 hours | -1) Advice + tailored booklet  2) Advice + generic booklet  3) No advice + tailored booklet  4) No advice + generic booklet = control  -Master's level clinicians  -Psychologist gave half-day seminars to review MI principles | -RCT  -Baseline, 3 and 12 months  -“Lifestyle assessment”, SIP and DrInC | -BIs in EDs may work best for those who attribute their injuries to alcohol  -Those with higher readiness to change had greater weekly alcohol consumption | -Higher readiness to change was related to increased alcohol consumption at follow-up  -Injured patients benefitted from BI  -Heighted emotional state at upon ED admission impacts BI effectiveness  -BI was more effective for women than men  -Age was not found to impact BI | -Engagement  -Increased self-efficacy  -Resolving ambivalence  -Increased insight/ awareness |
| Woolard et al. (2011)  High and Moderate | -N = varied by study  -Attrition varied by study  -ED locations varied by study  -Recruitment varied by study | -Young adults (18 to 65)  -Injuries and alcohol-related medical conditions | -BI: Described using FRAMES  -Suggested that it should be implemented by trained counselors | -Review article  -Designs varied by study (included RCTs and meta-analyses)  -Outcomes measures varied by study | -Mixed results: Some studies found BIs to be most effective in reducing alcohol-related injuries, rather than alcohol consumption  -Other studies found BIs led to reductions in consumption, and increased likelihood of accessing treatment | -Injured patients can benefit from BI  -Heighted emotional state upon ED admission positively impacts BI effectiveness  -Dependent drinkers may be less likely to benefit from BIs, and may require ongoing counselling (all suggested) | -Engagement  -Resolving ambivalence (tip decisional balance toward reducing alcohol consumption) |
| Wright, et al. (1998)  High and Weak | -N = 94  -13.9% refused/ dropped out at follow-up  -St. Mary’s AandE department, UK  -Convenience sample | -Ages 16+ (no mean reported)  -**%** female was not reported | **-**BI: Frames and referral to treatment (dependent on scores from screening)  -Control: None  **-**Alcohol Health Worker (AHW)  **-**Training was not reported | -Pre/post  -Baseline and 6 months  **-**SADQ | -Alcohol use significantly reduced at 6 months | -Severity of alcohol use impacts BI (suggested)  -Motivation to change upon ED admission will impact BI effectiveness (suggested) | -Resolving ambivalence |

*Note.* M = Mean; BNI = Brief Negotiated Interview; SBIRT = Screening, Brief Intervention and Referral to Treatment; NIAAA = National Institute of Alcohol Abuse and Alcoholism; AUDIT = Alcohol Use Disorders Identification Test; AUDIT-C = Alcohol Use Disorders Identification Test-Consumption; DrINC = Drinker Inventory of Consequences; SIP = Short Index of Problems; PAT = Paddington Alcohol Test; Form 90-AQ = Form 90-Alcohol Questionnaire; TLFB = Timeline Followback; ISS = Injury Severity Scale; MICS = Motivational Interviewing Skill Code; RTCQ = Readiness to Change Questionnaire; DIS = Diagnostic Interview Schedule; SADD = Short Alcohol Dependence Data; ASI = Alcohol Severity Index; RAPS4 = Rapid Alcohol Problems Screen-4; AIC = Adolescent Injury Checklist; ADQ = Adolescent Drinking Questionnaire; RAPI = Rutgers Alcohol Problems Inventory; SADQ = Severity of Alcohol Dependence Questionnaire.

^a^For more detailed outcomes, see Landy, Davey, Pecora, Quintero, and McShane (in progress). ^b^High/medium/low refers to the study’s relevance to the theory under study. High = provides insight regarding mechanisms and/or contextual factors of IPT. Medium = provided insight regarding either contextual factors or mechanisms. Low = No information on mechanisms or contextual factors were provided. Strong/moderate/weak refers to study’s rigour. Strong = randomized controlled trial (RCT), tightly controlled designs (e.g., validated measures of alcohol use and fidelity measures), appropriate statistical analysis and statistical information. Moderate: Usually pre-post designs with a balance between strengths and weaknesses. Weak = Lacking details (e.g., # of participants and attrition rate), inappropriate statistical analysis or lacking statistical information.
